# Cardio-Dense: Diagnosis of Cardiac Abnormalities Based on Phonocardiogram Using Improved Swin Transformer Through Lightweight Dense Blocks

**DOI:** 10.3390/diagnostics16101421

**Published:** 2026-05-07

**Authors:** Alaa E. S. Ahmed, Mostafa E. A. Ibrahim, Yassine Daadaa

**Affiliations:** College of Computer and Information Sciences, Imam Mohammad Ibn Saud Islamic University (IMSIU), Riyadh 11432, Saudi Arabia; asmohamed@imamu.edu.sa (A.E.S.A.); ymdaadaa@imamu.edu.sa (Y.D.)

**Keywords:** cardiovascular disorder, phonocardiography, continuous wavelet transform, deep learning, Swin transformers, DenseBlocks architecture, classification

## Abstract

**Background:** Cardiovascular diseases (CVDs) are among the top sources of mortality worldwide. To properly diagnose cardiovascular diseases, a low-cost remedy based on phonocardiography (PCG) signals must be proposed. Several deep learning (DL)-driven CVD systems are now being developed to identify various phases of the disease. Nevertheless, the approaches’ accuracy falls short of expectations, and they necessitate substantial processing resources and training data. **Methods:** This paper proposes Cardio-Dense, a hybrid framework for multi-class CVD detection from phonocardiogram signals. The PCG waveform is first denoised in the wavelet domain and then converted into a 2D time–frequency spectrogram using continuous wavelet transform (CWT). We design a joint architecture that combines a Swin transformer for capturing global contextual dependencies with lightweight DenseBlocks for efficient local feature refinement, enabling robust learning from PCG spectrograms across five disease classes. **Results:** Experiments on PCG datasets achieve up to 0.977 accuracy, 0.975 sensitivity, 0.992 specificity, 0.978 F1-score, 0.978 AUC, and 0.976 precision, while maintaining low computational overhead suitable for real-time inference. **Conclusions:** The findings indicate that the proposed model provides an economical, non-invasive method for preliminary signal-level identification of multi-class heart valve diseases. It benefits clinicians by decreasing the need for arduous and error-prone manual PCG analysis. Furthermore, it offers quick, near-real-time categorization suitable for clinical and portable applications.

## 1. Introduction

The World Health Organization (WHO) declared that, universally, CVDs are the prominent reason for loss of life, taking the lives of about 17 million people annually [1]. Other clinical problems with the heart, heart valves, or blood arteries are brought on by CVD disorders [2]. Thus, prompt evaluation in the early stages of life combined with evolving diagnostic methods may be able to reduce mortality rates [3], thereby averting many cases of death. There are several methods for detecting cardiac diseases, such as blood tests, angiography screening, electrocardiograms (ECGs) and phonocardiography. The visual depiction of a cardiac sound on a chart is called PCG, while the ECG is the electrical signal of heart movements. An ECG records the electrical impulses in the heart and is a common CVD screening tool [4]. Nevertheless, structural anomalies in heart valves are hard to detect using ECG waves [5]. Furthermore, several medical imaging technologies that can depict the cardiovascular system have been developed.

**Figure 1:**
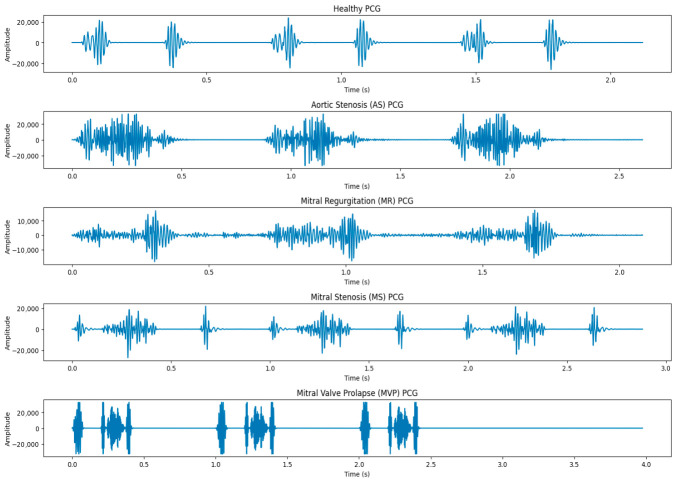
PCG signals of five CVD classes.

A visual record of the noises and murmurs produced by the contracting heart, including its valves and associated major vessels, can be obtained using the diagnostic method known as PCG [6]. It helps improve the diagnosis of cardiac disorders and develop fresh perspectives on the relationship between the signal and the heart’s mechanical operation. PCG can clearly diagnose a variety of cardio abnormalities such as aortic stenosis (AS), mitral stenosis (MS), mitral regurgitation (MR), and mitral valve prolapse (MVP). Figure 1 illustrates examples of these PCG signals divided into five classes. Observing and inspecting the PCG signal is laborious and prone to mistakes [7]. Yet substantial training and skill are needed to do the arbitrary PCG signal inspection and analysis that physicians require. This promotes the development of a computerized automated technique for anomaly identification from PCG signal cardiac assessment.

Research on the categorization of CVDs using biological signal processing and machine learning (ML) is currently showing promise. To detect heart valve diseases, machine learning methods are commonly exploited with PCG signals [8]. Regretfully, these methods’ precision is insufficient [9,10,11,12]. This is because machine learning algorithms rely on manual error-prone feature extraction schemes for instant amplitude, time interim, kurtosis, energy ratio, Mel-Frequency Cepstral Coefficients (MFCCs), and entropy. These metrics were frequently utilized in earlier research to perform dual categorization (normal PCG vs. aberrant PCG). However, they may not be sufficient for multiple-class categorization [10,11,12].

Different cardiovascular disorders reveal similar 1D signal properties. These similar linked properties may affect the multiple-class classification results. Consequently, it is critical to find out the discriminant features of different heart disease symptoms. Hence, classifying five levels of CVD using 1D PCG signals is challenging. Accordingly, to convert the energy of PCG signals to two-dimensional spectrograph images, this paper first exploits the discrete wavelet transform (DWT) for denoising the PCG input signals; second, it employs CWT to generate a 2D spectrograph.

Deep learning is extensively utilized in the detection and classification of many health disorders. It employs a sophisticated combination of feature-encoding algorithms to quickly learn from training data and produce reliable predictions. Deep learning models are widely used to extract deep features and classify heart sound waves into multiple classes [13,14].

This article introduces the Cardio-Dense system, which enhances cardiovascular disease diagnosis by combining a hierarchical multi-stage Swin transformer with a lightweight DenseBlock architecture, achieving a high accuracy, sensitivity, and specificity with affordable computational requirements.

### 1.1. Motivation and Research Questions

The limits of present diagnostic procedures and the worldwide burden of cardiovascular disease (CVD) are the driving forces behind this investigation. The particular elements consist of the following:

- With over 17 million deaths annually, cardiovascular disease is the world’s leading cause of death.
- Conventional visual screening of phonocardiogram (PCG) signals by medical practitioners is laborious, error-prone, and necessitates a great deal of specialized expertise.
- Achieving an accurate initial patient diagnosis is very important for cardiologists. There is a significant need for an affordable, non-invasive and robust computer-aided diagnostic tool in clinical settings.
- Although there are some deep learning-based systems, they frequently demand large training datasets, considerable processing power, and insufficient accuracy.
- A lot of earlier research primarily looked at binary classification (normal vs. aberrant). Because different cardiovascular diseases frequently share similar characteristics in one-dimensional data, it is challenging to classify PCG signals into five discrete stages.
- By creating a new hybrid architecture composed of a Swin transformer and convolutional DenseBlocks that can effectively and accurately classify five phases of heart valve illness using 2D spectrogram representations of PCG data, the study ultimately seeks to overcome these problems.

The research questions of this study are as follows:

- Can the Swin–DenseBlock hybrid model improve computing efficiency and diagnostic precision for the identification of cardiovascular diseases?
- To what extent may automated deep feature extraction be optimized by converting 1D heart sound samples into 2D spectrograms?
- Can five different heart valve problems be correctly classified without the use of hand-crafted characteristics or manual signal segmentation?

### 1.2. Research Contribution

To address the challenge of categorizing various CVDs from PCG signals, the authors use a novel deep learning approach. The article has made the following significant contributions:

- We present a powerful deep learning architecture that uses a hybrid Swin–Dense design to automate feature extraction and classification of CVDs from non-segmented PCG signals.
- A robust preprocessing pipeline is built, which employs DWT for noise suppression, followed by CWT to project 1D signals into a 2D time–frequency domain, preserving non-stationary cardiac features.
- The architecture innovatively combines Swin transformer blocks with DenseBlocks; which successfully captures long-range contextual relationships as well as fine-grained local morphological patterns.
- We developed a multi-scale cross-attention technique for feature fusion, allowing for efficient interactions between hierarchical representation and improved classification performance.
- The study presents a complete diagnostic framework capable of reliably categorizing heart sounds into five separate CVD classes, demonstrating the suggested model’s strong discriminatory power.
- The model maintained a good trade-off between accuracy and computational cost, making it suitable for practical application deployment settings.

### 1.3. Literature Review

This section summarizes the methodologies used to perform the computer-assisted sound cardiac diagnostic task. First, research studies employing machine learning techniques with hand-crafted feature extraction are discussed. Second, deep learning architectures utilized for the identification and classification of diverse CVDs are reviewed.

Cardiovascular disorder computer-assisted systems usually consist of several phases: (1) heart sound generation, (2) preprocessing, (3) segmentation, (4) feature engineering, and (5) classification. Heart sounds are generated using special sensors connected to the human body’s skin. The signals captured by the sensors are categorized in different forms such as ECG, pulse plethysmograph (PuPG), and PCG. These raw signals require preprocessing steps to remove noise signals. Next, segmentation, the process of determining and dividing the borders of murmurs and heart sounds, is applied. The segmentation stage is mostly used by ML algorithms, while DL models often rely on raw signals with a simple denoising scheme [14].

Important steps in the analysis of heart sound signals include feature mining and selection. Following denoising and segmentation, the original heart sound waves are often manually processed to acquire features. The literature has presented a wide range of feature extraction techniques. These features are categorized into time-based [8,15,16,17], frequency-based [18,19], and features which incorporate information in both the temporal and spectral domains [20]. Even though temporal–spectral-based features need more computational involvedness than temporal or spectral features, they can provide more comprehensive details about the PCG signal and superior feature extraction performance results [20]. Numerous transforms have been used to represent heart signals, such as S-transform, wavelet transform, and Hilbert transform. Making the distinction between aberrant and normal PCG signals is the aim of classification. Traditional machine learning algorithms, such as Gaussian Mixture Models (GMMs), random forests (RFs), Support Vector Machines (SVMs), Decision Trees (DTs), and K-Nearest Neighbor (KNN), usually integrate manually created features to accomplish tasks like classifying heart sounds [9,11,12,19,21,22].

In [9], the authors used two methods for feature extraction, discrete wavelet transform (DWT) and MFCCs, from two datasets of heart sounds. Then, they employed three ML models, RF, KNN and Extreme Gradient Boost (XGB), to classify the heart sound signals. Their results showed that RF resulted in the best accuracy of 88.7%. Despite its effectiveness, the approach relies on hand-crafted pictures, limiting its ability to capture complex PCG patterns and reducing robustness in noisy environments, thus motivating automated feature learning methods.

The researchers in [11] suggested using the imaginary component of the cross power spectral density (CPSD) to record the spectral range of heart sounds. With the aid of an ML framework, sub-band-based spectral characteristics derived from the imaginary components of CPSD were categorized. The system’s efficiency was assessed under conditions including the existence of white noise, automobile noise, or chatter. The preciseness of the suggested technique was 74.98%. However, their method did not account for physiological noise, limiting its effectiveness in real clinical settings and highlighting the need for more robust data-driven feature learning approaches.

An ML-based automatic diagnosis method for rheumatic heart disease (RHD) was presented in paper [12]. Using a free online dataset, heart sound data was gathered from 81 records of healthy controls (HC) and 124 additional records of people with RHD. To accurately depict RHD, thirty-one unique features made up of the temporal domain, frequency spectrum, perceptual domain, and acoustic domain were retrieved. Two nested cross-validation techniques were used to assess an SVM classifier, resulting in an F1-score of 96.0%. Yet the use of a traditional classifier and the lack of comparison with modern deep learning approaches limit its scalability and generalization across diverse clinical settings.

A thorough examination of several time and frequency domain features was conducted in paper [19]. They experimentally determined which feature subsets were useful for better categorization performance. Phonocardiogram signals, both segmented and unsegmented, were examined. The effectiveness of the MFCC features derived from unsegmented PCG and time–frequency characteristics of segmented data was assessed using a variety of ML and DL categorization algorithms, such as SVM, KNN, DT, ensemble classifier, artificial neural network (ANN), and long short-term memory (LSTM) networks. LSTM outperformed, with an AUC score of 91.39%. But the reliance on multiple feature–model combinations introduced increased complexity and limited the reproducibility and scalability of the approach.

A system designed for the identification and categorization of PCG signals into five classes was provided in [21]. First, a single-channel PCG signal was broken down into several modes named intrinsic mode functions (IMFs) via empirical mode decomposition (EMD). To generate a primary processed signal, they proposed an energy-based signal restoration method based on IMFs that was used to instantly locate and add the relevant IMFs. After being computed, the first nine MFCC features were submitted to various classification techniques, including ANN, Fine Tree, Ensemble Bagged Trees, Kernel Naive Bayes, Fine-KNN, and Quadratic Discriminant. Using 10-fold cross-validation and Fine-KNN, the greatest performance of 99.3% accuracy was attained. Meanwhile, in [22], they employed MFCCs and DWT for obtaining features from PCG waveforms, and they used SVM, deep neural networks (DNN), and centroid displacement-based KNN for learning and categorization. When the MFCC and DWT were combined, the outcomes were obviously better. With up to 97% accuracy, their method could diagnose patients with cardiac problems.

More importantly, ML methods’ precision was insufficient [9,11,12,19,21,22]. This is because machine learning algorithms rely on manual error-prone feature extraction schemes. Accordingly, deep learning architectures such as convolutional neural networks (CNNs) and LSTM were employed for CVD classification [10,23,24,25,26,27,28,29,30]. The researchers of [10] presented a technique that uses LSTM and temporal quasi-periodic properties to detect aberrant sounds. Short-Time Fourier Transform was used to extract features; afterwards, temporal quasi-periodic characteristics were computed, employing the mean magnitude difference. The PhysioNet/Cinc dataset was used for assessing effectiveness, and the findings demonstrated that their approach is comparable to state-of-the-art techniques. In [23], the authors extracted the spectrograms and MFCCs from two data sources of noisy heart signals. Then, they utilized many ML and DL models to classify the heart sound signals into five CVD classes. Their results showed that the Multi-Layer Perceptron (MLP) achieved the highest performance of 95.65% accuracy. However, the use of pre-extracted features and relatively simple architecture such as MLP may have limited the model’s ability to fully capture more complex patterns in PCG signals.

A technique for automatically diagnosing CVDs using PCG signals was proposed in [24]. The model involves tuning several hyperparameters, which can increase optimization complexity. They employed a CNN and power spectral Cardi-Net mixture while using a data enrichment technique; hence, they achieved an accuracy of 98.88%. The authors of [25] investigated different mixes of resolution and regularization techniques to improve the performance of DL-based CVD classification models. The LSTM and 1D-CNN DL models were assessed using the PCG dataset to classify five CVD types. A mixture of temporal and frequency features were extracted from the PCG signals using the Gabor dictionary. They obtained an accuracy of 98.95%. However, the model may involve relatively high computational cost, which could impact its efficiency in resource-constrained environments.

CardioXNet, a unique lightweight end-to-end CRNN design, was suggested in [26] for the automatic detection of five kinds of CVDs employing raw PCG signals. It comprised representation and sequence residual learning steps. During the representation learning phase, three concurrent CNN paths were constructed to obtain optimal time-independent features using a 2D-CNN-based squeeze–expansion. Due to the skip connection and bidirectional LSTMs, the network effectively extracted temporal features during the sequential residual learning phase without requiring any feature extraction on the data. The acquired findings showed a precision of 88.09% on a combined dataset comprising the Github PCG and PhysioNet datasets, and a correctness of up to 86.57% on the PhysioNet/CinC dataset.

The researchers of [27] created a unique attention-based method (CVT-Trans) to identify and classify PCG signals into five groups using a convolutional vision transformer. Representative characteristics were extracted from PCG data using the continuous wavelet transform-based spectrogram approach. The CVT-Trans system demonstrated an overall average accuracy (ACC) of 100% and an SE of 99.00% on a dataset of 1000 PCG signals. In [28], they identified the high-order spectral component of a phonocardiogram and suggested a preprocessing strategy for heart sound diagnostics. Additionally, to classify five CVDs, a multi-convolutional neural network (mCNN) is built. In order to determine the amplitude of the heart sound, the waveform of the audio file is segmented and stored as a picture using gray-scale treatment. Utilizing the magnitude information, the heart sound waves that are exceptionally noisy are filtered out. Following a 50% superposition separation of the heart audio recordings, picture data were saved and high-order spectrum features were obtained. The overall correctness for the five-category dataset was 99%.

The authors of [29] presented FHSU-NETR, a fetal heartbeat U-Net Transformer. It is a novel transformer-based deep learning model that was introduced for the automatic extraction of fetal heart rhythms in raw PCG signals. The model was verified on data collected from 20 healthy moms at Tohoku University Hospital’s prenatal outpatient clinic, and it was trained using an actual generated dataset. In [30], common features of the healthy and aberrant PCG signals were identified through the analysis of spectrograms based on the Short-Time Fourier Transform (STFT). To train the CNN model, spectrograms produced from the Pascal and PhysioNet datasets were used. The PhysioNet dataset yielded an accuracy of 95.4%, whereas the PhysioNet/Pascal combined dataset yielded an accuracy of 94.2%. The main issue of this CVD classification methodology is that it performs binary classification. Also, the use of stacked U-Net structures increases computational complexity, which may impact efficiency and scalability in practical applications. Table 1 describes recent related research efforts for the identification and classification of CVD.

The Swin transformer has been employed in many health applications such as the classification of retinal diseases [31], the classification of muscular dystrophies from MRI scans [32], and the detection of breast cancer from mammography images [33].

Sun et al. [31] introduced the PMP-Swin transformer, which employs a framework designed to obtain a precise classification of retinal diseases from fundus images. Traditional CNN-based methods often struggle to distinguish diseases with subtle differences in visual appearance, especially when multiple pathologies overlap in the same retinal region. The introduced Patch Message Passing (PMP) module allows local patches’ information to be shared across multiple scales. This design improves the model’s ability in capturing both fine-grained local details and broader contextual features. It also leverages the shifted window mechanism of the Swin transformer in handling large, high-resolution images in an efficient way. The model was tested on a new high-quality dataset called OPTOS, in addition to several public fundus datasets. It outperformed state-of-the-art models in multi-class retinal disease classification tasks.

The authors of [32], Okar et al., presented the use of the Swin transformer to classify muscular dystrophies from magnetic resonance imaging (MRI) scans. The model differentiates between Becker Muscular Dystrophy (BMD), Limb-Girdle Muscular Dystrophy type 2 (LGMD2), and healthy controls. Traditional CNNs have limitations in modeling long-range spatial dependencies, while the Swin transformer is able to capture relationships across distant regions of muscle tissue. This is important for identifying progressive degenerative patterns in MRI scans. The training procedure of the model is based on MRI contrasts, while focusing on fat fraction imaging. The model was compared to several CNN-based baselines, and the results indicated that the Swin transformer achieved better and more accurate classification (up to ~96%), even on relatively small datasets.

In the work of [33], the authors developed the MV-Swin-T (Multi-View Swin Transformer), which was employed for breast cancer detection from mammography images. Usually, mammography examinations typically give multiple views of each breast (e.g., craniocaudal and mediolateral oblique). These views are used to give an accurate diagnosis, which is a very important type of diagnosis. Conventional CNNs usually process each view independently, while MV-Swin-T is able to fuse information across different views using shifted-window dynamic attention blocks. One benefit of this model is to provide the ability to capture inter-view dependencies, which are considered as a more holistic illustration of breast tissue abnormalities. This work used CBIS-DDSM and VinDr-Mammo as datasets, and the results showed a higher classification performance compared to baseline CNN and single-view transformer models.

**Table 1:** A state-of-the-art comparison with limitations.

Ref.	Features	ML/DL Model	Dataset	Performance	#Classes	Limitations
[9]	DWT and MFCC	RF-MFO-XGB Ensemble	CHSPC	ACC.: 89%	Three	Depends on feature extraction and defines many parameters
[10]	Temporal quasi-periodic features using Short-Time Fourier Transform	LSTM	Physio-Net	Overall score: 94.48%	Two	Computationally expensive
[11]	Imaginary cross power spectral density (ICPSD)	SVM	PRV	ACC.: 74.98%	Two	Feature extraction only local but no focus on global features
[12]	Acoustic, frequency, time, perceptual domain	SVM+RBF	PRV + Physio-Net	F1-score: 96%	Two	Huge feature extraction
[19]	MFCC	LSTM + SVM + KNN + DT	Physio-Net	AUC: 91.39%	Two	Limited capability due to two classes
[22]	MFCC and DWT	SVM, DNN, and KNN	PRV	ACC.: 97%	Five	Several machine learning algorithms
[23]	MFCC	MLP	CHSPC	ACC.: 95.65%	Five	Limited capability due to two classes
[24]	Power Spectrum Density of PCG (PSD)	CNN	Physio-Net	ACC.: 98.88%	three	Defines huge hyperparameters
[25]	Gabor dictionaries of time–frequency atoms	CNN-LSTM	-	ACC.: 98.95%	Five	Computationally expensive
[26]	Time-invariant features	Lightweight CRNN	Yaseen [22] PhysioNet	ACC.: 99.6% ACC: 86.57%	Five	Limited features
[27]	Continuous wavelet transform spectrogram (CWTS)	Convolutional vision transformer (CVT)	Phonocardiogram dataset [22]	F1-score of 98%	Five	CVT has high computational complexity and resource demands
[28]	2D high-order spectral features	Multi-CNN (mCNN)	PhysioNet/CinC, Dataset of Yaseen [22]	ACC.: 99%	Five	Extensive computational resources and large amounts of labeled data
[29]	NA- Raw PCG signals	Pipeline of three 1D U-Nets	20 Healthy Pregnant Women Hospital of Tohoku University	Mean difference: 2.72 bpm	Two	Stacking of multiple U-Nets increased computational complexity
[30]	STFT-based spectrograms	CNN	PhysioNet/CinC PASCAL	ACC.: 95.4%	Two	High computational and memory requirements
[34]	NA- preprocessed (denoising, normalization, and down sampling) PCG signals	Convolution and Transformer Encoder Neural Network (CTENN)	PhysioNet/CinC Dataset of Yaseen [22] PRV	ACC.: 96.4%, ACC.: 99.7%, ACC.: 95%	Two Five Two	Significant computational complexity and resource demands

Accuracy: ACC, DWT: Discrete Wavelet Transform, MFCC: Mel-Frequency Cepstral Coefficients, RF: Random Forest, MFO: Moth Flame Optimization, XGB: Extreme Gradient Boost, CHSPC: Circulating Hematopoietic Stem/Progenitor Cell, LSTM: Long Short-Term Memory, SVM: Support Vector Machines, PRV: Private, RBF: Radial Basis Function, KNN: K-Nearest Neighbors, DT: Decision Tree, DNN: Deep Neural Network, MLP: Multilayer Perceptron, CNN: Convolutional Neural Network.

Based of the literature review, the following are the main research gaps that motivated our study:

- Systems capable of multi-class classification for particular cardiac valve illnesses are lacking because the majority of prior research has been on binary categorization (normal vs. abnormal) [10,11,12,19,29,30].
- Current machine learning models frequently rely on manually created features, which are subjective, time-consuming to design, and frequently insufficient for intricate multi-class recognition.
- The feasibility of current deep learning-based systems for real-time clinical applications is often limited by their need for large datasets and excessive processing capacity.
- While traditional attention methods suffer from quadratic complexity, which makes it challenging to effectively balance global and local data interactions, transformers frequently lack the inductive bias (such as shift and scale invariance) present in dense block convolutions.
- Current automated CVD detection technologies’ accuracy is still “up-to-the-mark” for accurate clinical diagnosis.

### 1.4. Paper Organization

The rest of the paper is structured as follows: The suggested approach, including the PCG dataset, preprocessing, CWT signal transformation into a 2D spectrogram, and the proposed Swin–DenseBlock model, is presented in Section 2. Section 3 presents the experimental protocol and the findings. Section 4 provides a thorough discussion of the attained results. Finally, Section 5 concludes the research study and provides the limitations of this research.

## 2. Materials and Methods

**Figure 2:**
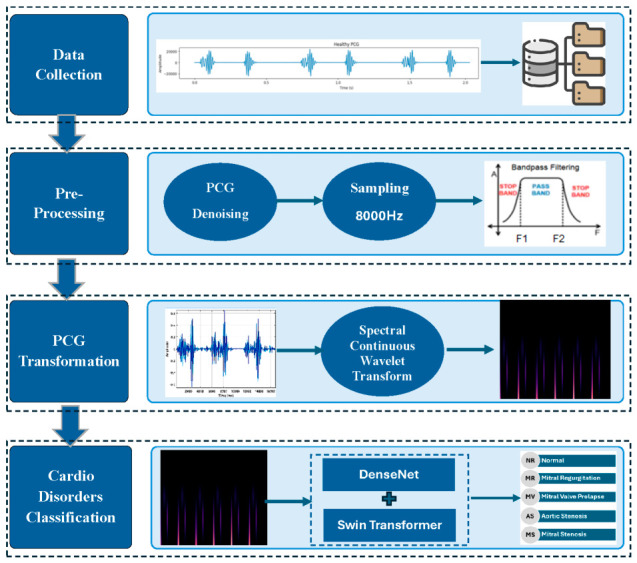
Cardio-Dense system flow diagram.

To investigate the categorization of cardiac sounds, without segmentation, into five unique categories—AS, MS, MR, MVP, and normal—this work offers a deep learning architecture called Swin–Dense architecture [35]. A CWT spectrogram is used to extract typical characteristics for the Cardio-Dense model, which detects abnormal patterns in PCG data. Using CWT, the PCG signal’s instantaneous power is first split up into different sub-bands. These sub-bands retain PCG’s oscillation properties and are used as discriminant features. Secondly, these features undergo a continuous wavelet transform-based spectrogram (CWTS) conversion from single-dimensional (1D) to two-dimensional (2D) spectrograms. Then, to classify the spectrograms into five distinct categories, the Swin–Dense model is developed by combining Swin transformer multi-stages with three parallel DenseBlocks. The architecture captures long-range contextual relationships (Swin transformer features) as well as fine-grained local morphological patterns (DenseBlock features). During the training phase, the hyper parameters of the architecture are continuously refined. Figure 2 shows a graphic representation of the Cardio-Dense architecture’s methodical phases. Using the readily accessible PCG datasets, multiple-class categorization (normal vs. AS vs. MR vs. MS vs. MVP) was carried out to evaluate the efficacy of the proposed technique. Lastly, the classification results are evaluated using a range of performance measures.

To summarize, the hybrid Swin–DenseBlock architecture provides an automated phonocardiogram categorization that combines a hierarchical Swin transformer backbone with multi-scale cross-attention and DenseBlock refinement. The Swin transformer extracts multi-scale features through efficient window-based self-attention. Meanwhile, the cross-attention layer combines Swin transformer Stage 3 and Stage 4 representations to improve discriminative capacity. The next section presents the data acquisition and preprocessing pipeline.

### 2.1. Data Acquisition and Preprocessing

The phonocardiogram dataset utilized in this study is the one referenced in [22]. The collection contains 1000 wav audio recordings, although the number of patients represented is unknown. There are no explicit patient identifiers or metadata provided in the dataset, so the analysis is conducted based on the signal level, not the patient level. The sampling frequency is 8000 Hz. The dataset includes five classes of heart sound signals (HSS): one normal class and four types of valvular heart diseases, MS, MVP, MR, and AS. Each category contains 200 audio recordings, resulting in a total of 1000 recordings across all five categories. Heart sound signals in the database have a duration ranging from 1.1556 to 3.9929 s. We utilize a unified recording time of 1.1556 s for the HSS, which is based on the dataset’s lowest HSS signal time. The original HSS dataset’s five categories can be found in the repository (https://github.com/yaseen21khan/Classification-of-Heart-Sound-Signal-Using-Multiple-Features- (accessed on 11 June 2025) [22]).

PCG signals are usually contaminated by noise from multiple sources. Filtering noise is therefore essential to get rid of these distortions. This should come at the cost of retaining all diagnostic information required for PCG signal processing while eliminating all noise-causing extraneous elements. In order to minimize background noise, the PCG signals undergo thorough filtering.

Heart sound transmissions generally have a frequency content that is focused between 20 and 150 Hz [28]. Pathological heart murmurs, however, might have frequency components that go beyond this range. Digital filtering is used to reduce noise and unimportant frequency components. A fourth-order Butterworth bandpass filter with cutoff frequencies of 15 Hz and 150 Hz is used in this work to analyze the heart sound signals, decreasing high-frequency noise and low-frequency drift while maintaining pathological significant information. The chosen range reflects a trade-off between signal fidelity and noise suppression. The five PCG classes are illustrated visually in Figure 1.

Wavelet-based denoising is a useful method for preprocessing phonocardiogram (PCG) signals and lowering noise. This technique separates signal components from noise by utilizing the discrete wavelet transform’s (DWT) multi-resolution analysis capability.

Wavelet denoising starts by decomposing the one-dimensional phonocardiogram signal $x\left(t\right)\in R^{N}$ into multiscale wavelet coefficients using the DWT:

(1) $$\begin{array}{cc} x\left(t\right)\to & \left\{a_{J},d_{1},\ldots ,d_{J}\right\} \end{array}$$

where $a_{J}$ denotes the approximation coefficient at level J, and $d_{J}$ is the detail coefficient at level J. The noise level is estimated from the finest-scale detail coefficients using the median absolute deviation (MAD):

(2) $$\hat{\sigma }=\frac{median\left(|d_{J}-median(d_{J})|\right)}{0.6745}$$

A threshold value λ is then calculated to remove the noise components from the wavelet coefficients. The universal threshold, given in [36], is defined as

(3) $$\lambda =\hat{\sigma }\sqrt{2\log N}$$

Soft thresholding is applied to each detail coefficient c to suppress noise:

(4) $$\begin{array}{c} \eta_{\lambda }(d_{j})=\left\{\begin{array}{cc} sign(d_{j})\left(|d_{j}|-\lambda \right), & |d_{j}|>\lambda \\ 0, & \mathrm{otherwise} \end{array}\right. \end{array}$$

This process minimizes noise while keeping the essential aspects of the signal. Finally, the denoised signal is regenerated by using inverse discrete wavelet transformations (IDWTs).

### 2.2. Data Augmentation

To enhance the model generalization and to address limitations in the size and variability of the utilized dataset, a carefully formulated data augmentation strategy was employed during the training phase. Despite being balanced between classes, the original dataset had relatively few samples, which could cause overfitting and decreased robustness when the model was exposed to new data. To address these issues, many augmentation techniques were used, such as temporal shifting, additive Gaussian noise, and amplitude scaling. Temporal shifting was performed by applying random translation to the signal across the time axis to replicate cardiac cycle alignment changes. By applying additive Gaussian noise, real-world acquisition noise is simulated, and the noise robustness is improved. Also, amplitude scaling is employed to capture and simulate the changes in signal intensity generated from the differences in both the sensor placement and amplitude scaling. The applied data augmentation strategies cause a significant enhancement in the training data diversity and allow the proposed model to be applied to a broader range of realistic signal variations. Consequently, this reduces overfitting and improves generalization performance capability. The data resulting from the augmentation process were expanded where each classification class includes around 600 samples, which produces 3000 samples across all five classes.

### 2.3. PCG Signal Transformation

A spectrogram is a common method in signal processing for time–frequency analysis because it shows how a signal’s frequency content changes over time [37]. This representation is particularly helpful for PCG data since several diagnostically significant patterns, including murmurs and transient components, may be weak or unclear in the raw time waveform. Prior PCG studies and reviews have noted that spectrogram-based analysis can highlight these frequency-dependent characteristics and support more reliable classification, particularly when combined with modern machine learning pipelines [38,39,40]. The process involves converting a 1D preprocessed PCG signal into a 2D time–frequency representation using the CWT. For short-time-varied PCG signals, CWT offers multiple levels of analysis (i.e., high time resolution at high frequencies and high frequency resolution at low frequencies), in contrast to the STFT approach, which has a fixed window.

The preprocessed PCG signal x(t)∈R is then transformed into a time–frequency representation using the following process. x(t)∈R denotes a time PCG signal. The CWT yields a complex-valued coefficient representation W(a,b)∈C, where R and C denote the sets of real and complex numbers, respectively. The CWT of *x*(*t*) is given by

(5) $$W(a,b)=\frac{1}{\sqrt{\left|a\right|}}\int_{-\infty }^{\infty }x(t)\;\psi^{*}(\frac{t-b}{a})dt$$

where x(t) is the denoised PCG signal, a is the scale (frequency parameter), b is time shift parameter, and $\psi^{*}(t)$ is the complex conjugate of the mother wavelet. To ensure that the energy of the wavelet remains constant across scales, a normalization factor is used: $\frac{1}{\sqrt{\left|a\right|}}$.

Because of its Gaussian-windowed complex sinusoid shape, which mimics the dampened oscillations of PCG signals, the mother wavelet is chosen. The mother wavelet is defined as follows:

(6) $$\psi (t)=e^{i\omega_{0}t}e^{\frac{t^{2}}{2}}$$

Equation (7) defines the relation between the pseudo-frequency $f_{a}$ and the scale parameter *a*:

(7) $$f_{a}=\frac{f_{c}.f_{s}}{a}$$

where $f_{c}$ is the center frequency of the mother wavelet ($\omega_{0}$ = 2 π $f_{c}$), and $f_{s}$ is the sampling frequency of the PCG signal.

The spectrogram is obtained by computing the magnitude-squared of the CWT coefficients:

(8) $$S(a,b)=|W(a,b){|}^{2}$$

where S(a,b) is the spectrogram over time.

**Figure 3:**
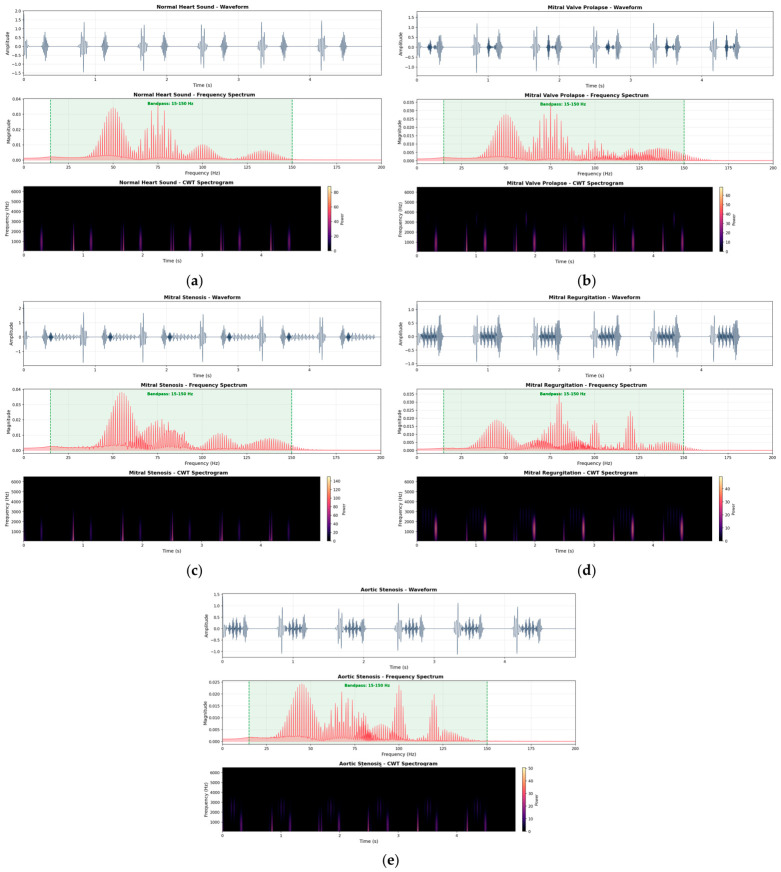
PCG signal processing, frequency spectrum and spectrogram transformation: (**a**) normal, (**b**) MVP, (**c**) MS, (**d**) MR, and (**e**) AS.

The conversion of PCG signals into time–frequency- and frequency-domain representations for various classes is shown in Figure 3. A typical sample of a cardiac raw PCG waveform is shown in each subfigure, together with the associated frequency spectrum and the generated spectrogram for the five categories: N, MVP, MS, MR, and AS. As demonstrated, diseased cases display clear spectral and temporal differences. These variations demonstrate how well CWT spectrogram-based representations capture discriminative characteristics, within the applied frequency band, for precise cardiovascular state classification. Finally, all the resulting spectrograms are resized to a unified 224 × 224 resolution using Bicubic interpolation.

### 2.4. Proposed Hybrid Architecture

**Figure 4:**
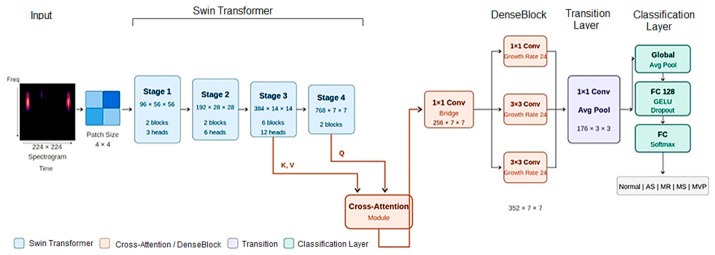
The Cardio-Dense system’s layer-by-layer architecture for classifying cardiovascular disorders.

For PCG spectrogram classification, the proposed hybrid Swin–DenseNet architecture with lightweight cross-attention incorporates several processing stages designed to capture both local discriminative information and global context. The design of the proposed system’s layer architecture is shown in Figure 4.

The input PCG spectrogram be represented as a matrix **X** ∈ ℝ^(T×F×1)^, where T and F denote the time and frequency dimensions, respectively (typically 224 × 224 after CWT transformation). The spectrogram is partitioned into N non-overlapping patches of size P × P (where P = 4), yielding N = (T/P) × (F/P) = 56 × 56 = 3136 patches. Each patch x*_i_* ∈ ℝ^P2^ is flattened and projected into a D-dimensional latent space (D = 96) using a linear embedding layer followed by layer normalization:

(9) $$E_{i}=\mathrm{LayerNorm}\left(W_{E}\cdot \mathrm{flatten}\left(x_{i}\right)+b_{E}\right)$$

where E*_i_* ∈ ℝ^D^ denotes the embedded representation of the *i*-th patch.

Several recent studies have shown that the Swin transformer can balance computational efficiency with good performance across a variety of computer vision tasks, which has led to its widespread use in the literature [31,32,33,35].

Hence, the Swin transformer serves as the primary feature extractor, capturing hierarchical and global contextual information from the input spectrograms.

The embedded patches are processed through a four-stage Swin transformer backbone, where each stage applies shifted window multi-head self-attention (SW-MSA) to capture local and cross-window dependencies. The hierarchical structure progressively reduces spatial resolution while increasing channel depth:

(10) $$Z^{\left(s\right)}={\mathrm{SwinStage}}_{s}(Z^{\left(s-1\right)}),s\in \{1,2,3,4\}$$

The four hierarchical stages have stage depths of [2, 2, 6, 2], attention heads of [3, 6, 12, 24], and output dimensions of 96 × 56 × 56, 192 × 28 × 28, 384 × 14 × 14, and 768 × 7 × 7. A patch merging layer that concatenates features from 2 × 2 neighboring patches and applies linear projection for down sampling is used between stages.

To enhance feature interactions across different hierarchical levels, a lightweight multi- scale cross-attention mechanism fuses the high-resolution features of Stage 3 with the semantically rich features of Stage 4. This operation emphasizes informative multi-scale relationships while maintaining computational efficiency:

(11) $$Q=W_{Q}\cdot Z^{\left(4\right)},\;K=W_{K}\cdot Z^{\left(3\right)},\;V=W_{V}\cdot Z^{\left(3\right)}$$

(12) $$Z_{\mathrm{cross}}=\mathrm{softmax}\left(\frac{QK^{⊤}}{\sqrt{d_{k}}}\right)V+Z^{\left(4\right)}$$

where **W***_Q_* ∈ ℝ^(768×768)^ and **W***_K_*, **W***_V_* ∈ ℝ^(768×384)^ are learnable projection matrices, and *d_k_* is the per-head dimension. The residual connection preserves Stage 4 features while enriching them within the multi-scale context.

The cross-attention output is projected through a 1 × 1 convolution bridge to reduce channel dimensionality:

(13) $$Z_{\mathrm{bridge}}=\mathrm{GELU}(\mathrm{BN}({\mathrm{Conv}}_{1\times 1}(Z_{\mathrm{cross}})))$$

where **Z**_bridge_ ∈ ℝ^(256×7×7)^.

The bridged features are then processed through three parallel convolutional DenseBlocks with a growth rate k equal to 24:

(14) $$Z_{\mathrm{dense}}=\mathrm{Concat}\left[Z_{\mathrm{bridge}},B_{1}(Z_{\mathrm{bridge}}),B_{2}(Z_{\mathrm{bridge}}),B_{3}(Z_{\mathrm{bridge}})\right]$$

where B_1_ is a 1 × 1 (Conv, BN, ReLU) block of 24 output channels, and B_2_ and B_3_ are two 3 × 3 (Conv, BN, ReLU) blocks of 24 output channels, yielding Z_dense_ ∈ ℝ^(352×7×7)^.

DenseBlocks are employed to perform efficient local feature refinement and feature reuse through dense connectivity. Strong and reliable classification performance is produced by this hybrid architecture, which combines effective local feature extraction with global modeling capacity. A transition layer applies channel compression, with a factor θ equal to 0.5, and spatial down sampling, with average pooling 2 × 2 for the batch normalized $Z_{\mathrm{dense}}$, yielding Z_trans_ ∈ ℝ^(176×3×3)^.

The refined features are then aggregated using global average pooling (GAP) to obtain a fixed-length representation:

(15) $$z_{\mathrm{global}}=\mathrm{GAP}(Z_{\mathrm{trans}})=\frac{1}{H\times W}\sum_{h,w}Z_{\mathrm{trans}}^{\left(h,w\right)}$$

where **z**_global_ ∈ ℝ^176^.

Finally, to accurately identify and classify the five cardiac disorder classes, the pooled feature vector is passed through a two-layer fully connected classifier with non-linear activation and regularization, while a SoftMax function is applied to the output logits to obtain the projected class probabilities.

(16) $$\begin{array}{l} h=\mathrm{Dropout}(\mathrm{GELU}(W_{1}\cdot z_{\mathrm{global}}+b_{1})),\\ \hat{y}=\mathrm{Softmax}(W_{2}\cdot h+b_{2}) \end{array}$$

where W_1_ ∈ ℝ^(128×176)^, W_2_ ∈ ℝ^(C×128)^, and C = 5 is the number of target classes (normal, AS, MR, MS, MVP).

Model training minimizes the cross-entropy loss with optional label smoothing (ε):

(17) $$L=-\frac{1}{N}\sum_{n=1}^{N}\sum_{c=1}^{C}{\tilde{y}}_{n}^{\left(c\right)}log({\hat{y}}_{n}^{\left(c\right)})$$

where the smoothed target is

(18) $${\tilde{y}}_{n}^{\left(c\right)}=(1-\varepsilon )\cdot y_{n}^{\left(c\right)}+\frac{\varepsilon }{C}$$

Optimization is performed using the AdamW optimizer with a learning rate η = 10^−4^, weight decay λ = 10^−4^, and mixed-precision training for computational efficiency. Early stopping with patience of eight epochs monitors the validation macro-F1-score. Table 2 illustrates the utilized hyperparameters along with their typical values.

## 3. Experimental Results

### 3.1. Experimental Protocol

The phonocardiogram dataset utilized in all experiments is the one referenced in [22], which is outlined in Section 2.1. To guarantee uniform input length across all samples, each HSS was resampled to 8000 Hz and truncated to a predetermined fixed time before training. Every recording underwent the same process of feature extraction and model inference.

A stratified 10-fold cross-validation method was employed to offer a trustworthy and objective evaluation of the proposed architecture. To prevent data leakage, all data splits were performed at the level of the original PCG recordings prior to any data augmentation. The original dataset of 1000 recordings was distributed as 200 samples per class. These data are divided into ten stratified folds, each with 100 samples and a balanced class distribution of 20 samples per class. For each iteration, eight folds were used for training, one fold was used as the test set, and the subsequent fold was used as the validation set. Data augmentation was only applied to the training set, increasing the number of original samples from 800 to 2400 in order to preserve assessment integrity. There were no changes made to the test and validation sets.

**Figure 5:**
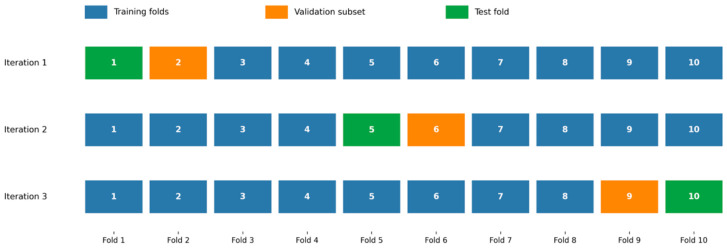
Visualization of the stratified 10-fold cross-validation protocol.

The model was evaluated on the original test fold after being trained on the supplemented training data and verified on the original validation fold for early halting and model selection. To guarantee that every sample was used precisely once for testing, this procedure was performed for each of the ten folds. This methodology allows for accurate model performance estimation, balanced class representation in each fold, and the strict separation of training and evaluation data. Figure 5 summarizes the cross-validation pipeline. It is worth mentioning that the dataset labels are mutually exclusive, and the proposed model is not trained or evaluated with hybrid heart abnormalities (i.e., case of multi-label outputs). Each preprocessing step applied to the PCG signals is deterministic and performed separately within each cross-validation fold to ensure reproducibility and prevent data leaks. The dataset is initially split into training, validation, and testing subsets using the stratified 10-fold cross-validation process; no data from the full dataset is used prior to splitting. Normalization is applied at the individual signal level rather than using global statistics. Initially, the dataset is divided into three subsets, training, validation and testing, using a 10-fold cross-validation procedure. Only the training subset is subjected to data augmentation; the testing and validation sets are left unaltered. These design choices ensure an unbiased, reliable, and fair evaluation of the proposed model. To guarantee equitable and repeatable evaluation, the same hyperparameter configuration is used for every fold.

The proposed method is implemented on a standard machine equipped with an Intel Core i7 10th generation processor and 32 GB of RAM. All quantitative results reported in this study are based exclusively on stratified 10-fold cross-validation.

### 3.2. Results Analysis

**Figure 6:**
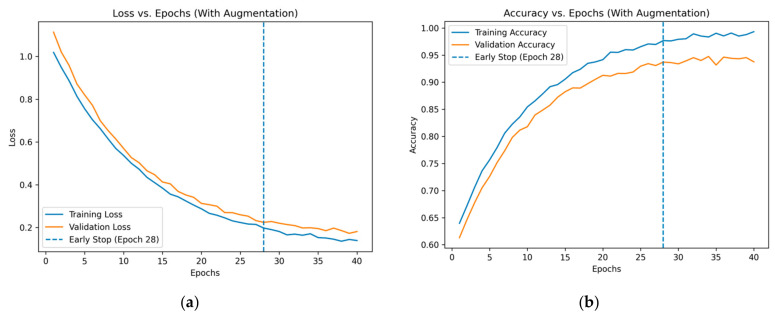
Training and validation (**a**) loss and (**b**) accuracy curves of the proposed model over 40 epochs.

The learning curves in Figure 6 illustrate the training and validation performance of the proposed model with data augmentation used for training over 40 epochs. As shown in the loss curve, both training and validation losses decrease steadily during the early epochs, indicating effective learning and convergence. The validation loss follows a similar trend to the training loss, with only a small gap between them, suggesting reduced overfitting due to the applied augmentation strategy. The early stopping criterion indicates that training and validation losses start to stabilize at epoch 28. To avoid overfitting and enhance generalization, early stopping is used, which ends training when the validation loss stops improving for a certain number of epochs.

This behavior is further supported by the accuracy curves, which show a consistent increase in training and validation accuracy throughout training. Strong generalization capability is demonstrated by the model’s high validation accuracy and narrow generalization gap relative to training accuracy. Overall, these findings demonstrate how well the augmentation technique works to increase model robustness, avoid overfitting, and promote steady convergence.

**Figure 7:**
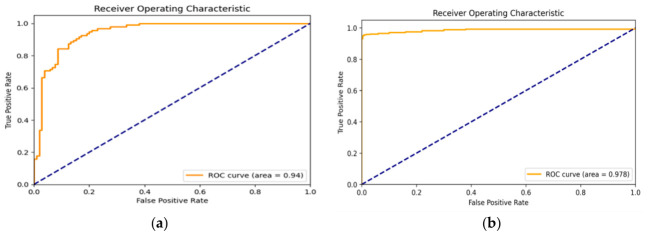
A visual diagram of receiver operating characteristic (ROC) AUC. (**a**) The proposed system without preprocessing step, and (**b**) the proposed system with preprocessing step.

Figure 7 presents a visual representation of the receiver operating characteristic (ROC) area under the curve (AUC) with two distinct scenarios. Panel (a) illustrates the AUC for the proposed system without any preprocessing steps, showcasing the system’s performance in its raw form. On the other hand, panel (b) shows the AUC for the proposed system, after incorporating a preprocessing step. This comparison visually highlights the impact of preprocessing on the system’s efficiency, indicating the improvements gained by introducing this additional step in the Cardio-Dense system’s workflow.

**Figure 8:**
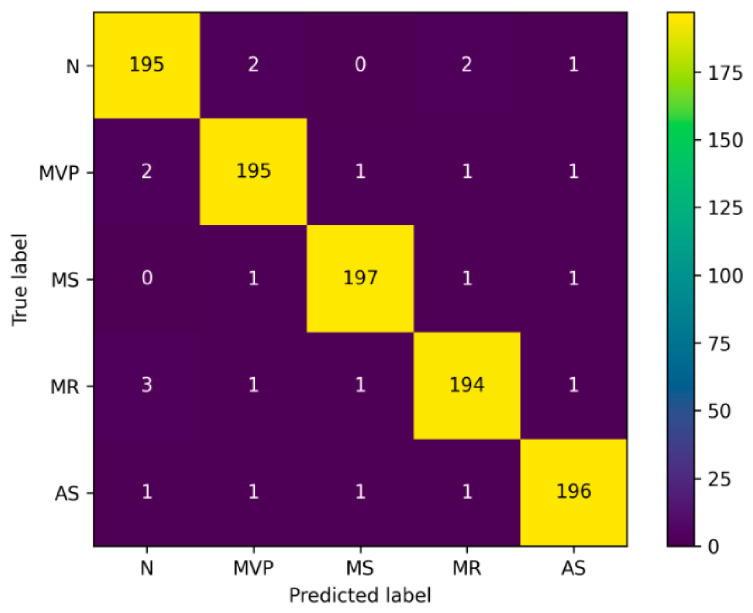
Aggregated confusion matrix across stratified 10-fold cross-validation.

Figure 8 presents the aggregated confusion matrix illustrating the classification performance of the proposed Cardio-Dense model across five heart sound categories: AS, MR, MS, MVP, and normal PCG signals.

The confusion matrix is generated from out-of-fold test predictions aggregated across the stratified 10-fold cross-validation protocol. This ensures that each of the 1000 recordings contributes precisely once to the assessment. A fair evaluation of class-wise discriminating performance is made possible by the equal representation of each class with 200 recordings.

The number of correctly classified recordings for each class is represented by the diagonal elements of the matrix, whilst misclassifications between classes are represented by the off-diagonal elements. All categories show strong diagonal dominance, revealing that the model detects most heart sound signal patterns in a correct way. Misclassifications are limited and relatively evenly distributed, with no single class showing a dominant confusion pattern. Overall, the confusion matrix demonstrates that the Cardio-Dense model effectively discriminates among the five heart sound categories, with a low rate of classification errors under signal-level evaluation.

#### 3.2.1. Classification Results

In this section, the classification results of the five classes are presented. The classification per class performance of the proposed Cardio-Dense framework across the five heart sound categories is summarized in Table 3. The reported results reflect the macro-average values calculated across all ten folds instead of focusing on a single best-performing run. Given the small size of the dataset, this procedure offers a more reliable and objective assessment of classification performance.

The consistently high sensitivity, specificity, precision, F1-score, and accuracy indicate that the model is effective at discriminating pathological and normal heart sound signal patterns under the adopted signal-level evaluation protocol.

These findings show the proposed architecture’s potential as a computational tool for automated heart sound analysis and show that it can learn robust representations from phonocardiogram data.

To evaluate the statistical significance of the performance obtained with data augmentation, a paired t-test was conducted on the cross-validation results. Specifically, performance metrics such as accuracy and F1-score were computed across ten folds for both augmented and non-augmented settings. The results show that the proposed Cardio-Dense model achieved an average accuracy of 0.977 ± 0.006 with augmentation, compared to 0.961 ± 0.002 without augmentation, as shown in Table 4. The paired t-test yielded a p-value less than 0.01, indicating that the observed improvement is statistically significant. This demonstrates that the augmentation method improves the generalization performance and robustness of the model.

The per-fold performance of the proposed model is demonstrated in Table 5. The performance is measured over stratified 10-fold cross-validation. The results confirm the model’s stability and dependability, which show consistent behavior across folds with just slight variations.

#### 3.2.2. Comparison to Baseline Architectures

Table 6 compares various deep learning models for cardiovascular disease categorization, utilizing SE, SP, F1-score, precision, ACC, and AUC. Conventional convolutional designs like VGG16 and VGG19 have reasonable performance, with accuracy values of 0.938 and 0.945, respectively. DenseNet121 enhances the results by achieving an accuracy of 0.955 through efficient feature reuse. The Swin transformer provides a comparable performance, with an accuracy of 0.953 and the ability to capture global contextual information via self-attention. Our proposed model performs the best and most consistently across all evaluation parameters, with an SE of 0.975, SP of 0.992, F1-score of 0.978, precision of 0.976, ACC of 0.977, and AUC of 0.978. This improvement is due to the successful integration of Swin transformer-based global feature extraction, cross-attention-based feature refining, and DenseBlock-driven local feature learning.

#### 3.2.3. Comparison with State-of-the-Art Models

**Figure 9:**
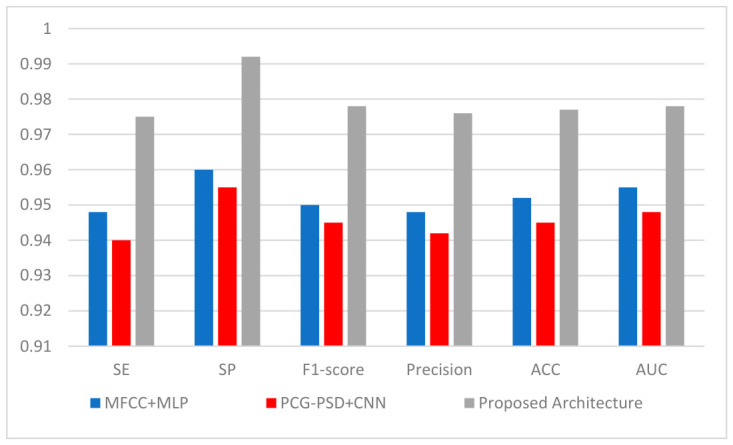
Comparison with reimplemented state-of-the-art models on the same dataset, MFCC+MLP [23], PCG-PSD+CNN [24] and proposed architecture.

Figure 9 compares the proposed Cardio-Dense model to selected state-of-the-art approaches reimplemented using the same dataset and evaluation protocol. As demonstrated, the suggested model consistently outperforms the compared techniques of [23,24] on all assessment metrics. The proposed model has a higher sensitivity, specificity, F1-score, precision, accuracy, and AUC, indicating that it can successfully capture both global and local properties of PCG signals. The enhancement is due to the combination of Swin transformer-based global feature extraction, cross-attention-based feature fusion, and DenseBlock-driven local feature refining. Overall, the results show that the proposed approach improves classification accuracy and reliability under persistent experimental settings.

#### 3.2.4. External Validation Using Independent Dataset

The PhysioNet/CinC 2016 dataset was used for external validation in order to further evaluate the suggested model’s capacity for generalization. A popular publicly accessible heart sound dataset for assessing automated phonocardiogram (PCG) categorization systems is the PhysioNet/CinC 2016 dataset. It is made up of 3126 to 3240 wav-format audio files that were collected from 1072 patients in a variety of clinical and environmental settings, including both normal and pathological heart sounds. The dataset is especially useful for evaluating model resilience because it shows considerable variety in recording quality, noise levels, and acquisition devices. The dataset in this work is used for external evaluation to determine how well the suggested model can generalize during domain shift. The classification job is designed as a binary problem, differentiating between normal and pathological heart sound recordings [41], in order to be compatible with this dataset.

It is anticipated that performance decreases in comparison to the results attained across validation and testing with the Yassine [22] dataset due to variations in data distribution, recording settings, and label definitions. On the external independent dataset PhysioNet/CinC 2016 [41], the suggested model demonstrated good robustness under domain shift, with an accuracy of roughly 0.88 and an AUC of 0.91 utilizing the augmented dataset for training, while the results were lower when no augmentation was used, as shown in Table 7, which consistently demonstrates the augmentation’s efficacy in improving generalization. Validating our proposed model using the independent dataset PhysioNet/CinC 2016 (Table 7) provides preliminary proof of its generalizability.

#### 3.2.5. Complexity Analysis

The computational complexity of the implemented hybrid Swin–Dense architecture is examined using asymptotic notation as a function of input spectrogram dimensions in order to guarantee hardware-independent evaluation. N = H × W represents the total number of spatial tokens in the input spectrogram with spatial resolution H × W.

CWT is used in the time–frequency transformation stage, where the number of scales S determines the additional multiplicative factor that CWT requires, yielding a complexity of O(S⋅NlogN).

The four backbone Swin transformer stages that significantly lower the spatial resolution are used to extract global features. Per-stage complexity is obtained by computing self-attention inside non-overlapping local windows of fixed size M × M. The per-stage complexity can be expressed as $O\left(\frac{N}{\alpha_{s}}\cdot M^{2}\cdot d_{s}\right)$, where $\alpha_{s}$ represents the spatial reduction factor at stage s, and $d_{s}$ denotes the embedding dimension at that stage. This formulation avoids the quadratic complexity $O(N^{2})$ associated with global self-attention mechanisms.

To fuse multi-scale features, a cross-attention mechanism is introduced between feature maps at different resolutions. Let $N_{i}$ and $N_{j}$ denote the token counts of the two feature levels. The cross-attention complexity is given by $O\left(N_{i}\cdot N_{j}\cdot d_{k}\right)$, where $d_{k}$ is the attention projection dimension. This operation captures inter-scale dependencies while maintaining reduced computational cost due to hierarchical resolution.

The convolutional bridge and DenseBlock components contribute additional complexity through convolution operations, expressed as $O\left(N_{j}\cdot k^{2}\cdot C_{in}\cdot C_{out}\right)$, where k is the kernel size, and $C_{in}$ and $C_{out}$ represent the input and output channel dimensions, respectively. The DenseBlock structure further enhances feature propagation through densely connected layers and controlled channel growth, followed by a transition layer with compression factor θ.

Finally, the overall total per-sample inference complexity is approximated as

(19) $$O(NlogN)+O\left(\sum_{s}\frac{N\cdot M^{2}\cdot d_{s}}{\alpha_{s}}\right)+O(N_{i}\cdot N_{j}\cdot d_{k})+O(N_{j}\cdot k^{2}\cdot C)$$

This formulation shows that the architecture preserves rich multi-scale feature representations while maintaining quasi-linear scaling with input resolution. Furthermore, practical efficiency is assessed using parameter count, floating-point operations (FLOPs), and inference latency under defined conditions (input resolution: 224 × 224, single-channel specification). Computational complexity is compared to the different bassline architectures in Table 8.

**Figure 10:**
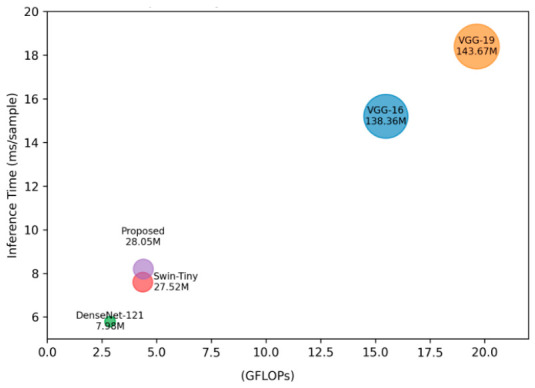
Computational complexity comparison (bubble size ≡ number of parameters).

The Swin transformer backbone accounts for 98.1% of the 28.05 million trainable parameters in the proposed architecture, while the cross-attention module, DenseBlock, and classification head together account for the remaining 1.9%. With the cross-attention and DenseBlock components adding less than 1% more FLOPs, the computational cost of 4.39 GFLOPs each forward pass is still similar to the baseline Swin-Tiny (4.36 GFLOPs). An average inference latency of 8.2 ms per sample is shown in measurements performed on the GPU, indicating that it is suitable for real-time clinical application. On the test dataset, the peak GPU memory use is roughly 2.5 GB during mixed-precision training. Because fully connected layers account for 89% of parameters, VGG structures have a significant computational overhead of 15–20 GFLOPs, as seen in Table 1. With comparable FLOPs to Swin-Tiny and improved feature refinement via multi-scale cross-attention and dense connection, the suggested hybrid Swin–Dense achieves a favorable trade-off between representational capacity and computational efficiency. Figure 10 presents a visual representation of the computational complexity comparison with the baseline architectures.

## 4. Discussion

Cardiovascular diseases are considered one of the major global causes of death and require the presence of effective and precise diagnostic technologies. Since the process of interoperating phonocardiogram signals is traditionally done on a manual basis, the results can be laborious and prone to human error. Developing accurate and efficient diagnostic tools is achieved by developing automated systems that use cutting-edge machines and deep learning techniques.

The preprocessing step is essential for improving the quality of PCG signals, in which noise and artifacts are excluded. In this paper, we preprocessed PCG signals using a combination of wavelet denoising and bandpass filtering. This method successfully lowers noise while preserving important characteristics, preparing the signals for further examination. The continuous wavelet transform-based spectrogram provides a rich time–frequency representation and is used to convert the preprocessed signals into 2D spectrograms.

The Swin transformer architecture is employed because it uses shifted window-based self-attention techniques to capture global contextual information. The Swin transformer effectively extracts pertinent characteristics by portioning the input spectrograms into non-overlapping patches and using linear embeddings. This architecture is very suitable for studying complex PCG signals because of its hierarchical nature, which enables the attainment of both local and global patterns.

To improve feature extraction capabilities, we integrated DenseBlocks with the Swin transformer. The DenseBlocks’ connectivity reduces the vanishing gradient issue and allows effective feature reuse. Additionally, the feature maps are further refined by passing the output of the Swin transformer through DenseBlocks to extract minute information, which is essential for producing a precise classification. This integration produces a robust and reliable feature extraction pipeline.

The experiment’s results demonstrate the effective and consistent performance of the proposed model across all evaluation metrics. Table 6 shows that the model outperforms baseline designs such as VGG16, VGG19, DenseNet121, and the Swin transformer, with SE = 0.975, SP = 0.992, F1 = 0.978, precision = 0.976, ACC = 0.977, and AUC = 0.978. The resulting improvement achieved confirms that a positive interaction occurred between convolutional feature refinement and hierarchical self-attention. From an architectural perspective, the Swin transformer’s incorporation enables an efficient modeling of global contextual interactions through localized self-attention, while the DenseBlock component strengthens local feature extraction and enhances feature reuse. Additionally, the model can detect both high-level and fine-grained discriminative patterns in PCG signals by successfully integrating multi-level representations through the application of cross-attention. The consistent performance improvements over both purely convolutional and transformer-based baselines are explained by the suggested approach.

The per-fold results shown in Table 5 demonstrate the robustness of the proposed model. As shown in the cross-validation table, the model shows little variation across folds, with only slight fluctuations around the mean values. This stability indicates that the learned representations generalize well across different data splits and are not sensitive to specific training subsets. The narrow variability across folds supports the reliability of the reported performance and reduces the likelihood of overfitting. This stability shows that the learnt representations are not sensitive to training subsets and generalize effectively across various data splits. The low variability among folds lessens the possibility of overfitting and supports the claimed performance’s dependability.

A further understanding of classification behavior is provided by the confusion matrix analysis. The confusion matrix analysis in Figure 8 provides additional insight into classification behavior. The model achieves high true positive rates across all classes, with only limited misclassification between structurally similar disorders.

The proposed model is compared with two recent methods that have been reimplemented using the same dataset and assessment procedure in Figure 9. As shown, for all evaluation measures, the proposed model consistently performs better than the methods of [23,24]. Conducting additional validation on an independent dataset, PhysioNet/CinC 2016 (Table 7), provides preliminary evidence of generalization for our proposed model.

From a computational standpoint, the suggested model performs better, while maintaining a complexity profile similar to the Swin transformer. The employment of cross-scale attention and effective DenseBlock structures guarantees that the overall computational cost stays acceptable even though the hybrid design adds new components. This implies that efficiency and accuracy are well balanced, which makes the model suitable for deployment scenarios in the real world. The complexity of the proposed architecture is furthermore analyzed using a number of model parameters, floating point operations, and inference time per sample, as shown by Table 8 and the bubble chart visualization in Figure 10.

Overall, the findings show that the proposed architecture offers a reliable and efficient method for PCG signal-level heart valve disorder classification, attaining primary evidence of generalization, high accuracy, and a fair trade-off between computational economy and performance.

Cardio-Dense is designed not to be a replacement to cardiologists; it is considered a clinical decision support system. In the real world, this system can be used as a complementary tool for cardiologists to have an automated and accurate pre-scanning of heart signal recordings. Furthermore, Cardio-Dense is suitable to be integrated and deployed in point-of-care environments such as outpatient clinics. Also, it is suitable to be deployed in telemedicine to help in giving reliable assessments. Overall, the multivariant uses of the system should be kept and used under the supervision of medical professionals.

## 5. Conclusions

This paper proposed a Cardio-Dense, hybrid Swin–Dense architecture for automated phonocardiogram classification, which combines hierarchical Swin transformer features with dense convolutional refinement. The proposed framework performed well across all evaluation metrics, achieving 97.7% accuracy, 97.5% sensitivity, 99.2% specificity, and 97.8% macro-averaged F1-score on a five-class cardiac disorder classification task that included normal, aortic stenosis, mitral regurgitation, mitral stenosis, and mitral valve prolapse categories. The design retains computational efficiency, with 28.05 million parameters and 4.39 GFLOPs, allowing for real-time inference at around 8.2 milliseconds per sample. The incorporation of DenseBlock feature refinement and cross-attention fusion between Stage 3 and Stage 4 representations enhances discriminative capability and at the same time minimizes computational overhead. The experimental results show consistent generalization throughout 10-fold cross-validation, indicating robustness to data partitioning.

These results show that the proposed paradigm presents an affordable, non-invasive method for the preliminary signal-level diagnosis of five heart valve disorders. It helps clinicians by reducing reliance on laborious and error-prone manual PCG analysis. Additionally, it provides fast, near-real-time classification appropriate for clinical and portable applications. The researchers identified several limitations within the current scope of the work, despite its achieved high accuracy:

- The shortage of large-scale, publicly accessible clinical datasets for heart sound classification causes serious limitations.
- Few datasets offer diverse labels for different heart sound categories. Most databases currently in use concentrate on binary classification—normal vs. abnormal—rather than multi-class scenarios.

## Figures and Tables

**Table 2:** Hyperparameters and their typical values.

Hyperparameter	Description	Value
Learning Rate (α)	Step size for weight updates	1 × 10^−4^ (AdamW optimizer)
Batch Size	Number of samples per iteration	32
Training Epochs	Maximum training iterations	40
G	L2 regularization factor	1 × 10^−4^
Dropout Rate	Regularization in classifier	0.2
Patch Size	Input patch size for Swin	4 × 4
Window Size	Local attention window	7 × 7
Embedding Dimension	Initial feature dimension	96
Transformer Depth	Number of blocks per stage	[2, 2, 6, 2]
Attention Heads	Multi-head attention config	[3, 6, 12, 24]
DenseBlock Layers	Number of conv layers	4
Growth Rate	Channel increase per layer	24

**Table 3:** Classification results of the proposed Cardio-Dense system.

Classes	SE *	SP *	F1-Score	Precision	ACC *	AUC *
N	0.975	0.995	0.980	0.982	0.978	0.975
MVP	0.975	0.993	0.978	0.975	0.976	0.975
MS	0.985	0.996	0.985	0.987	0.980	0.978
MR	0.970	0.987	0.965	0.955	0.975	0.970
AS	0.980	0.995	0.982	0.982	0.978	0.975

* Sensitivity: SE, specificity: SP, accuracy: ACC, receiver operating characteristic (ROC) area under the curve: AUC.

**Table 4:** Performance of the proposed model (with/without) data augmentation (mean ± SD over stratified 10-fold cross-validation), showing statistically significant enhancement of *p* < 0.01.

Dataset	Augmentation	Accuracy	F1-Score	AUC
Yassine [22]	No	0.961 ± 0.002	0.965 ± 0.003	0.962
Yassine [22]	Yes	0.977 ± 0.006	0.978 ± 0.005	0.978

**Table 5:** Ten-fold classification results.

Fold	SE	SP	F1	Precision	ACC	AUC
1	0.972	0.991	0.976	0.974	0.975	0.976
2	0.976	0.993	0.979	0.977	0.978	0.979
3	0.974	0.992	0.977	0.975	0.976	0.977
4	0.978	0.994	0.980	0.978	0.979	0.980
5	0.973	0.991	0.977	0.975	0.976	0.977
6	0.977	0.993	0.979	0.977	0.978	0.979
7	0.975	0.992	0.978	0.976	0.977	0.978
8	0.979	0.994	0.981	0.979	0.980	0.981
9	0.974	0.991	0.977	0.975	0.976	0.977
10	0.976	0.993	0.979	0.977	0.978	0.979

**Table 6:** Performance comparison to different baseline models.

Algorithm	SE	SP	F1	Precision	ACC	AUC
VGG16	0.928	0.948	0.938	0.930	0.938	0.935
VGG19	0.935	0.955	0.945	0.936	0.945	0.940
DenseNet121	0.948	0.965	0.955	0.948	0.955	0.950
Swin	0.952	0.970	0.960	0.952	0.953	0.952
Proposed Model	**0.975**	**0.992**	**0.978**	**0.976**	**0.977**	**0.978**

**Table 7:** Cross-validation with PhysioNet/CinC 2016 dataset.

Dataset	Augmentation	ACC	F1-Score	AUC
PhysioNet/CinC 2016 [41]	No	0.83	0.81	0.86
PhysioNet/CinC 2016 [41]	Yes	0.88	0.87	0.91

**Table 8:** Computational complexity comparison.

Model	Parameters (M)	FLOPs (G)	Inference (ms)
VGG-16	138.36	15.47	15.2
VGG-19	143.67	19.63	18.4
DenseNet-121	7.98	2.87	5.8
Swin-Tiny	27.52	4.36	7.6
Proposed	28.05	4.39	8.2

## Data Availability

The original HS dataset’s five categories can be found in the repository https://github.com/yaseen21khan/Classification-of-Heart-Sound-Signal-Using-Multiple-Features- (accessed on 11 June 2025).

## References

[1] World Health Organization (2021). A Report About Health.

[2] Soares L., Leal T., Faria A.L., Aguiar A., Carvalho C. (2023). Cardiovascular Disease: A Review. Biomed. J. Sci. Tech. Res..

[3] Alkhodari M., Hadjileontiadis L.J., Khandoker A.H. (2024). Identification of congenital valvular murmurs in young patients using deep learning-based attention transformers and phonocardiograms. IEEE J. Biomed. Health Inform..

[4] Khan A.H., Hussain M., Malik M.K. (2021). Cardiac disorder classification by electrocardiogram sensing using deep neural network. Complexity.

[5] Molcer P.S., Kecskes I., Delić V., Domijan E., Domijan M. (2010). Examination of formant frequencies for further classification of heart murmurs. IEEE 8th International Symposium on Intelligent Systems and Informatics.

[6] Altaf A., Mahdin H., Alive A.M., Ninggal M.I.H., Altaf A., Javid I. (2023). Systematic Review for Phonocardiography Classification Based on Machine Learning. Int. J. Adv. Comput. Sci. Appl..

[7] Khan M.U., Samer S., Alshehri M.D., Baloch N.K., Khan H., Hussain F., Kim S.W., Zikria Y.B. (2022). Artificial neural network-based cardiovascular disease prediction using spectral features. Comput. Electr. Eng..

[8] Xu W., Yu K., Ye J., Li H., Chen J., Yin F., Xu J., Zhu J., Li D., Shu Q. (2022). Automatic pediatric congenital heart disease classification based on heart sound signal. Artif. Intell. Med..

[9] Rath A., Mishra D., Panda G., Pal M. (2022). Development and assessment of machine learning based heart disease detection using imbalanced heart sound signal. Biomed. Signal Process. Control.

[10] Zhang W., Han J., Deng S. (2019). Abnormal heart sound detection using temporal quasi-periodic features and long short-term memory without segmentation. Biomed. Signal Process. Control.

[11] Pathak A., Samanta P., Mandana K., Saha G. (2020). An improved method to detect coronary artery disease using phonocardiogram signals in noisy environment. Appl. Acoust..

[12] Asmare M.H., Filtjens B., Woldehanna F., Janssens L., Vanrumste B. (2021). Rheumatic heart disease screening based on phonocardiogram. Sensors.

[13] Ahmed A.E., Abbas Q., Daadaa Y., Qureshi I., Perumal G., Ibrahim M.E. (2023). A residual-dense-based convolutional neural network architecture for recognition of cardiac health based on ECG signals. Sensors.

[14] Chen J., Guo Z., Xu X., Jeon G., Camacho D. (2024). Artificial intelligence for heart sound classification: A review. Expert Syst..

[15] Ibrahim N., Jamal N., Sha’abani M.N.A.H., Mahadi L.F. (2021). A comparative study of heart sound signal classification based on temporal, spectral and geometric features. 2020 IEEE-EMBS Conference on Biomedical Engineering and Sciences (IECBES).

[16] Singh S.A., Majumder S., Mishra M. (2019). Classification of short unsegmented heart sound based on deep learning. 2019 IEEE International Instrumentation and Measurement Technology Conference (I2MTC).

[17] Soares E., Angelov P., Gu X. (2020). Autonomous learning multiple-model zero-order classifier for heart sound classification. Appl. Soft Comput..

[18] Khaled S., Fakhry M., Esmail H., Ezzat A., Hamad E. (2022). Analysis of training optimization algorithms in the NARX neural network for classification of heart sound signals. Int. J. Sci. Eng. Res..

[19] Khan F.A., Abid A., Khan M.S. (2020). Automatic heart sound classification from segmented/unsegmented phonocardiogram signals using time and frequency features. Physiol. Meas..

[20] Cheng X., Wang P., She C. (2020). Biometric identification method for heart sound based on multimodal multiscale dispersion entropy. Entropy.

[21] Talal M., Aziz S., Khan M.U., Ghadi Y., Naqvi S.Z.H., Faraz M. (2023). Machine learning-based classification of multiple heart disorders from PCG signals. Expert Syst..

[22] Yaseen, Son G.Y., Kwon S. (2018). Classification of heart sound signal using multiple features. Appl. Sci..

[23] Abbas S., Ojo S., Al Hejaili A., Sampedro G.A., Almadhor A., Zaidi M.M., Kryvinska N. (2024). Artificial intelligence framework for heart disease classification from audio signals. Sci. Rep..

[24] Khan J.S., Kaushik M., Chaurasia A., Dutta M.K., Burget R. (2022). Cardi-Net: A deep neural network for classification of cardiac disease using phonocardiogram signal. Comput. Methods Programs Biomed..

[25] Fakhry M., Gallardo-Antolín A. (2024). Elastic net regularization and gabor dictionary for classification of heart sound signals using deep learning. Eng. Appl. Artif. Intell..

[26] Shuvo S.B., Ali S.N., Swapnil S.I., Al-Rakhami M.S., Gumaei A. (2021). CardioXNet: A novel lightweight deep learning framework for cardiovascular disease classification using heart sound recordings. IEEE Access.

[27] Abbas Q., Hussain A., Baig A.R. (2022). Automatic Detection and Classification of Cardiovascular Disorders Using Phonocardiogram and Convolutional Vision Transformers. Diagnostics.

[28] Li Y., Yi J., Zhong B., Yi Z., Chen A., Jin Z. (2024). Heart Sounds Classification Based on High-Order Spectrogram and Multi-Convolutional Neural Network after a New Screening Strategy. Adv. Theory Simul..

[29] Almadani M., Alkhodari M., Ghosh S.K., Khandoker A.H. (2023). FHSU-NETR: Transformer-Based Deep Learning Model for the Detection of Fetal Heart Sounds in Phonocardiography. 2023 Computing in Cardiology (CinC).

[30] Khan K.N., Khan F.A., Abid A., Olmez T., Dokur Z., Khandakar A., Chowdhury M.E.H., Khan M.S. (2021). Deep learning based classification of unsegmented phonocardiogram spectrograms leveraging transfer learning. Physiol. Meas..

[31] Sun Y., Liu Y., Chen Y., Wang Y., Chen H. (2023). PMP-Swin: Multi-Scale Patch Message Passing Swin Transformer for Retinal Disease Classification. arXiv.

[32] Mastropietro A., Casali N., Taccogna M.G., D’Angelo M.G., Rizzo G., Peruzzo D. (2024). Classification of Muscular Dystrophies from MR Images Improves Using the Swin Transformer Deep Learning Model. Bioengineering.

[33] Sarker S., Sarker P., Bebis G., Tavakkoli A. (2024). MV-Swin-T: Mammogram Classification with Multi-View Swin Transformer. arXiv.

[34] Cheng J., Sun K. (2023). Heart Sound Classification Network Based on Convolution and Transformer. Sensors.

[35] Baima N., Wang T., Zhao C.-K., Chen S., Zhao C., Lei B. (2023). Dense Swin Transformer for Classification of Thyroid Nodule. 2023 45th Annual International Conference of the IEEE Engineering in Medicine & Biology Society (EMBC).

[36] Donoho D.L., Johnstone I.M. (1994). Ideal spatial adaptation by wavelet shrinkage. Biometrika.

[37] Oh S.L., Jahmunah V., Ooi C.P., Tan R.-S., Ciaccio E.J., Yamakawa T., Tanabe M., Kobayashi M., Acharya U.R. (2020). Classification of heart sound signals using a novel deep WaveNet model. Comput. Methods Programs Biomed..

[38] Popalzai P.K., Khattak K.S., Sohail A.M., Khan Z.H. (2025). Enhancing Cardiac HealthDiagnoses Through Machine Learning Analysis of Phonocardiograms (PCG). J. Data Sci. Intell. Syst..

[39] Zhu B., Zhou Z., Yu S., Liang X., Xie Y., Sun Q. (2024). Review of Phonocardiogram Signal Analysis: Insights from the PhysioNet/CinC Challenge 2016 Database. Electronics.

[40] Dwivedi A.K., Imtiaz S.A., Rodriguez-Villegas E. (2018). Algorithms for automatic analysis and classification of heart sounds—A systematic review. IEEE Access.

[41] Liu C., Springer D., Moody B., Silva I., Johnson A., Samieinasab M., Sameni R., Mark R., Clifford G.D. (2016). Classification of Heart Sound Recordings: The Physionet Computing in Cardiology Challenge 2016.

